# Antimicrobial Activities of *Dictyostelium* Differentiation-Inducing Factors and Their Derivatives

**DOI:** 10.3390/biom9050163

**Published:** 2019-04-27

**Authors:** Yuzuru Kubohara, Yoshiko Shiratsuchi, Hirotaka Ishigaki, Katsunori Takahashi, Yoshiteru Oshima, Haruhisa Kikuchi

**Affiliations:** 1 Laboratory of Health and Life Science, Graduate School of Health and Sports Science, Juntendo University, Inzai, Chiba 270-1695, Japan; 2 Department of Molecular and Cellular Biology, Institute for Molecular and Cellular Regulation (IMCR), Gunma University, Maebashi 371-8512, Japan; 3 Department of Health Sciences, School of Health and Social Services, Saitama Prefectural University, Koshigaya, Saitama 343-8540, Japan; shiratsuchi-yoshiko@spu.ac.jp; 4 Department of Medical Technology, Faculty of Health Science, Gunma Paz College, Takasaki 370-0006, Japan; ishigaki@paz.ac.jp (H.I.); k-takahashi@paz.ac.jp (K.T.); 5 Laboratory of Natural Product Chemistry, Graduate School of Pharmaceutical Sciences, Tohoku University, 6-3, Aza-aoba, Aramaki, Aoba-ku, Sendai 980-8578, Japan; oshima@mail.pharm.tohoku.ac.jp (Y.O.); hal@mail.pharm.tohoku.ac.jp (H.K.)

**Keywords:** antimicrobial, *Dictyostelium discoideum*, DIF, MRSA, VRE

## Abstract

At the end of its life cycle, the cellular slime mold *Dictyostelium discoideum* forms a fruiting body consisting of spores and a multicellular stalk. Originally, the chlorinated alkylphenone differentiation-inducing factors (DIFs) -1 and -3 were isolated as stalk cell inducers in *D. discoideum*. Later, DIFs and their derivatives were shown to possess several biologic activities including antitumor and anti-*Trypanosoma* properties. In this study, we examined the antibacterial activities of approximately 30 DIF derivatives by using several bacterial species. Several of the DIF derivatives strongly suppressed the growth of the Gram-positive bacteria *Staphylococcus aureus*, *Bacillus subtilis*, and *Enterococcus faecalis* and *Enterococcus faecium*, at minimum inhibitory concentrations (MICs) in the sub-micromolar to low-micromolar range. In contrast, none of the DIF derivatives evaluated had any noteworthy effect on the growth of the Gram-negative bacterium *Escherichia coli* (MIC, >100 µM). Most importantly, several of the DIF derivatives strongly inhibited the growth of methicillin-resistant *S. aureus* and vancomycin-resistant *E. faecalis* and *E. faecium*. Transmission electron microscopy revealed that treatment with DIF derivatives led to the formation of distinct multilayered structures consisting of cell wall or plasma membrane in *S. aureus*. The present results suggest that DIF derivatives are good lead compounds for developing novel antimicrobials.

## 1. Introduction

**Figure 1:**
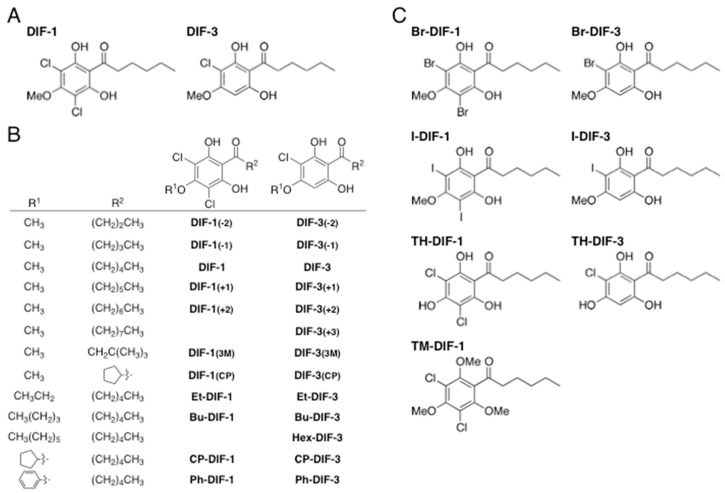
(**A**) Chemical structures of *Dictyostelium discoideum* differentiation-inducing factor (DIF) 1 and DIF-3. (**B**, **C**) Chemical structures of DIF derivatives used in this study.

The cellular slime mold *Dictyostelium discoideum* has long been studied as an excellent model system in the fields of cell and developmental biology; at the end of its developmental process, this organism forms fruiting bodies, each consisting of spores and a multicellular stalk [1,2,3]. Differentiation-inducing factor 1 (DIF-1) (Figure 1A) is a chlorinated polyketide that induces stalk cell differentiation [4,5] and modulates cell chemotaxis during the development of *D. discoideum* [6]. DIF-3 (Figure 1A) is a metabolite of DIF-1 and has virtually no biologic activity in *D. discoideum* [5,6,7,8].

In addition to these developmental roles, DIF-1, DIF-3, and several of their derivatives (Figure 1B,C) exert multiple biologic activities [9]. In particular, several derivatives demonstrate strong anti-proliferative and anti-metastatic activities in tumor cells in vitro and in vivo [10,11,12,13,14,15,16,17,18,19,20,21,22]. Some DIF derivatives increase glucose consumption in non-transformed mammalian cells in vitro and in vivo [23,24,25], and others show immunomodulatory effects in T cells in vitro [26,27,28]. Furthermore, we found several DIF derivatives that display strong in vitro and in vivo effects against *Trypanosoma cruzi*, the protozoan parasite that causes Chagas disease (human American trypanosomiasis) [29]. Importantly, modifying the side chains of DIF derivatives might provide a means to isolate individual biologic activities [16,22,23,24,26,27,28], such that these biologics might become candidate lead compounds for developing novel anti-cancer, anti-diabetic, immunomodulatory, and anti-*Trypanosoma* drugs [9]. Moreover, the results of previous studies lead us to expect that some DIF derivatives may possess as yet unidentified biologic effects.

In this study, we investigated the antibacterial activities of DIF derivatives in vitro and show that several of them exert strong antibacterial effects against Gram-positive bacteria, including *Staphylococcus aureus*, *Bacillus subtilis*, *Enterococcus faecalis* and, *Enterococcus faecium*. In addition, several of the DIF derivatives evaluated strongly inhibited the growth of methicillin-resistant *S. aureus* and vancomycin-resistant enterococci (i.e., strains of *E. faecalis* and *E. faecium*). Our results support the investigation of such DIF derivatives as candidate lead compounds for developing novel antimicrobials.

## 2. Materials and Methods

### 2.1. Bacterial Species and Reagents

The Gram-positive bacteria methicillin-susceptible *S. aureus* (MSSA; strains 209P and ATCC29213), methicillin-resistant *S. aureus* (MRSA; MS29202 and ATCC43300), vancomycin-susceptible *E. faecalis* (VSE; ATCC29212), vancomycin-resistant *E. faecalis* (VRE: VanB; ATCC51299), vancomycin-resistant *E. faecium* (VRE: VanA; ATCC700221), and *B. subtilis* (ATCC6633) and the Gram-negative bacteria *Escherichia coli* (NIHJ) and *Mycobacterium bovis* (PG45; ATCC25523) were used in this study. DIF derivatives (Figure 1) were synthesized as previously described [16,22] and stored at −20 °C as 10 mM solutions in DMSO or ethanol (EtOH). AB0022A, Pf-1, and Pf-2 were synthesized as described [30,31] and stored at −20 °C as 10 mM solutions in DMSO. A combined solution of penicillin (5000 units/mL) and streptomycin (5 mg/mL) was purchased from Gibco (ThermoFisher Scientific, Waltham, MA, USA); vancomycin and oxacillin were obtained from Sigma-Aldrich (St. Louis, MO, USA); and cefoxitin and ampicillin were bought from Wako Pure Chemical Industries (Osaka, Japan). The hydrophobic index (ClogP) of each DIF derivative was calculated by using ChemDraw 16.0 software (PerkinElmer, Inc. Waltham, MA, USA).

### 2.2. Paper-Disc Diffusion Assay

We mixed 0.1 mL of *S. aureus* suspension (10^9^ CFU/mL) in Mueller–Hinton broth (MHB) (0.2% (*w/v*) beef extract, 1.75% (*w/v*) casein hydrolysate, 0.15% (*w/v*) starch) into 10 mL of MHB agar (MHB containing 1.7% (*w/v*) agar; final bacterial density, 10^7^ CFU/mL) and spread this suspension in a plastic culture dish (diameter, 100 mm). Paper discs (diameter, 8 mm; Advantec, Tokyo, Japan) impregnated with 2 µL of EtOH, DMSO, a 10 mM solution of the DIF or DIF derivative of interest in EtOH or DMSO (approx. 5.5–8 µg DIF/disc), or the purchased penicillin–streptomycin solution (10 units penicillin and 10 µg streptomycin/disc) were placed on the bacterial agar plate. In addition, paper discs (KB disc ‘EIKEN’; EIKEN Chemical Company, Tokyo, Japan) containing minomycin (30 µg/disc), imipenem (10 µg/disc), gentamicin (10 µg/disc), levofloxacin (5 µg/disc), or clindamycin (2 µg/disc) were placed on a MHB bacterial agar plate as controls. The plates were incubated for 20 h at 37 °C, after which the bacterial growth-inhibition zones around each disc was noted.

In addition, a diffusion assay without paper discs was performed for comparison. Briefly, 10 mL of *S. aureus* in MHB (10^7^ CFU/mL) was spread on an MHB agar plate (diameter, 100 mm), after which 2 µL of each 10 mM DIF solution was dropped directly on the agar surface. The plates were incubated for 20 h at 37 °C, after which the bacterial growth-inhibition zones around the discs were noted.

### 2.3. Measurement of Minimum Inhibitory Concentration

Gram-positive and Gram-negative bacteria in MHB (5 × 10^5^ CFU/mL; 0.1 mL/well) were incubated for 20 to 24 h at 37 °C in 96-well plates (Corning, NY, USA) in the presence of vehicle, various concentrations of serially diluted DIF derivatives, or known antibiotics; MIC was defined as the lowest concentration of the additives that inhibited visible bacterial growth.

*M. bovis* was grown in Hayflick modified PPLO (pleuropneumonia-like organisms) broth (0.5% (*w/v*) glucose, 5% (*w/v*) beef heart infusion, 2.5% (*w/v*) yeast extract, 1% (*w/v*) peptone, 0.5% (*w/v*) NaCl, 15% (*v/v*) heat-inactivated horse serum, 50 µg/mL of ampicillin) and used at concentrations of 10^4^ to 10^7^ CFU/mL for the minimum inhibitory concentration (MIC) assay as described; tiamulin (Santa Cruz Biotechnology, Dallas, TX, USA) and enrofloxacin (Sigma-Aldrich) were used as positive-control antibiotics.

### 2.4. Transmission Electron Microscopy

Specimens for transmission electron microscopy were prepared according to standard procedures [32]. Briefly, *S. aureus* (strain ATCC29213) in MHB (1–5 × 10^8^ CFU/mL; 2 mL per well) was incubated for 1.5 h at 37 °C in 6-well plates (Corning) in the presence of 0.2% DMSO (vehicle) or 4 µM DIF derivative. After incubation, the bacteria were transferred to centrifuge tubes, collected by centrifugation (4000 × *g*, 1 min), fixed overnight at 4 °C in 0.5 mL of 2.5% (*v/v*) glutaraldehyde in 100 mM phosphate buffer (pH 7.4) (LSI Medience Corporation, Tokyo, Japan), and postfixed in 1% (*w/v*) OsO_4_ in 100 mM phosphate buffer. Fixed specimens were dehydrated through a graded series of ethanol and embedded in Epon812 (Oken-Shoji, Tokyo, Japan). Ultrathin sections were cut by using an ultramicrotome (model UC6; Leica, Wetzlar, Germany) and stained with uranyl acetate and lead citrate. These sections were examined under a transmission electron microscope (model HT7700; Hitachi High-Tech, Tokyo, Japan).

## 3. Results

### 3.1. Antimicrobial Effects of DIFs and Their Derivatives on S. aureus in Agar Plates

**Figure 2:**
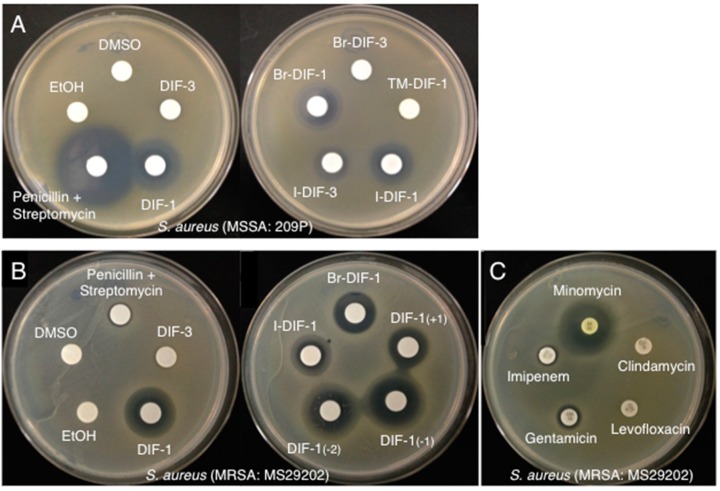
(**A**) Effects of DIF derivatives on the growth of methicillin-susceptible *Staphylococcus aureus* (MSSA) on agar. Paper discs were placed on Mueller–Hinton broth (MHB) agar plates that contained MSSA (209P); paper discs were impregnated with 2 µL of either ethanol (EtOH; vehicle), DMSO (vehicle), a 10 mM solution of the indicated DIF or DIF derivative, or a mixture of penicillin (5000 units/mL) and streptomycin (5 mg/mL). After incubation for 20 h at 37 °C, bacterial growth-inhibition zones around the discs were noted. (**B**, **C**) Effects of DIF derivatives on the growth of methicillin-resistant *S. aureus* (MRSA) on agar. Paper discs were placed on MHB agar plates that contained MRSA (MS29202); these discs were impregnated with 2 µL of either EtOH (vehicle), DMSO (vehicle), a 10 mM solution of the indicated DIF or DIF derivative, or a mixture of penicillin and streptomycin (**B**). In addition, paper discs containing the indicated antibiotics were placed on MRSA agar plates (**C**). After incubation for 20 h at 37 °C, bacterial growth-inhibition zones around the discs were noted.

We first examined the effects of DIF-1, DIF-3 (Figure 1A), and their derivatives (Figure 1B,C) on the growth of methicillin-susceptible *S. aureus* (MSSA: strain 209P) (Figure 2A) and methicillin-resistant *S. aureus* (MRSA: strain MS29202) (Figure 2B) by using a paper-disc diffusion assay. In MSSA (Figure 2A), a large ring free of bacterial growth formed around the disc containing penicillin and streptomycin, as expected. Similar growth-free rings developed around the discs containing DIF-1 and several DIF-1 derivatives but not DIF-3. In MRSA-containing plates (Figure 2B), penicillin and streptomycin failed to inhibit bacterial growth. However, DIF-1 and several DIF-1 derivatives, but not DIF-3, again produced zones free of bacterial growth. Although the MRSA strain we used was susceptible to minomycin, it was resistant to imipenem, levofloxacin, and clindamycin and was marginally resistant to gentamicin (Figure 2C). These results indicate that various DIFs and their derivatives possess antimicrobial activity against *S. aureus*. In addition, the mechanism underlying the antimicrobial action of DIFs likely differs (at least in part) from those of the known antibiotics that we used.

### 3.2. MIC Values of DIF Derivatives in S. aureus, B. subtilis, E. coli, and M. bovis

We next examined the effects of DIF derivatives on the growth of the Gram-positive bacteria MSSA (strain 209P), MRSA (MS29202), and *B. subtilis* (ATCC6633) and on the Gram-negative bacterium *E. coli* (NIHJ), and MIC values of DIF derivatives were determined (Table 1). Several of the DIF derivatives showed strong antibacterial activity against the Gram-positive bacteria, yielding MIC values of less than 1 µM. However, none of the DIF derivatives that we tested inhibited the growth of *E. coli* and *M. bovis* (MIC, >100 µM) (Table 1); *M. bovis* was sensitive to the antibiotics tiamulin (MIC, 0.5 µM) and enrofloxacin (0.7 µM).

**Figure 3:**
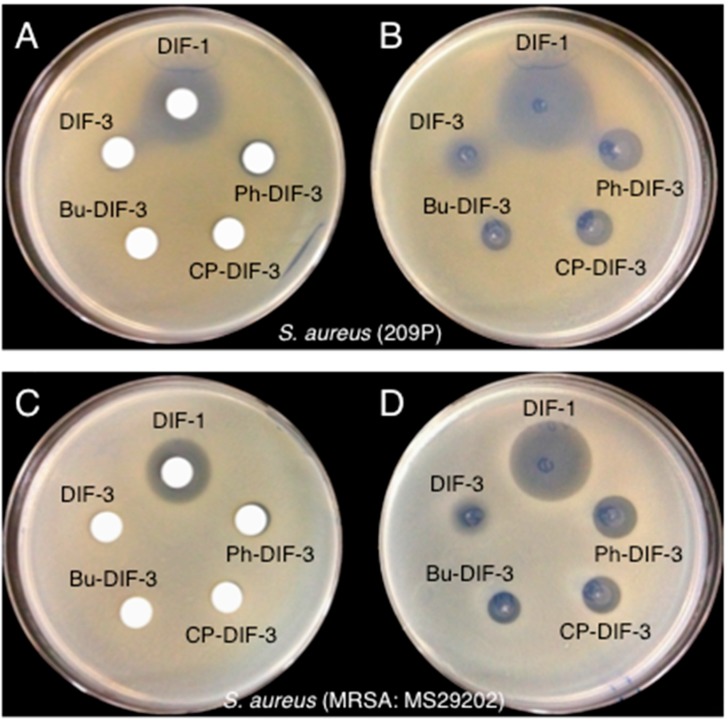
Effects of DIF derivatives on the growth of MSSA and MRSA on agar. Paper discs were placed on MHB agar plates that contained (**A**) MSSA (strain 209P) or (**C**) MRSA (MS29202); each paper disc was impregnated with 2 µL of a 10 mM solution of the indicated DIF derivative. In parallel, 2 µL of a 10 mM solution of the indicated DIF derivative was placed directly on MHB agar plates that contained (**B**) MSSA (209P) or (**D**) MRSA (MS29202). After incubation for 20 h at 37 °C, bacterial growth-inhibition zones around the discs were noted.

Note that the MIC values of DIF-3 against MSSA and MRSA were comparable to those of DIF-1 (Table 1), even though DIF-3 lacked antibacterial activity against these strains in the paper-disc diffusion assay (Figure 2). These results suggest that DIF-3 and its derivatives exert antibacterial activity in solution but become ineffective on solid media (e.g., paper disc, agar). To examine this hypothesis, we compared the effects of DIF-1, DIF-3, and three DIF-3 derivatives on the growth of MSSA and MRSA by using DIF-impregnated paper discs or by spotting the DIF solutions directly on the surface of the bacteria-containing agar (Figure 3). Again, DIF-1—but not DIF-3 or its derivatives—showed a strong antibacterial activity against MSSA and MRSA in the paper-disc diffusion assay (Figure 3A,C). However, when the solutions were dropped directly on the agar, all of the DIF derivatives we tested showed antibacterial activity against the bacteria (Figure 3B,C). These results suggest that DIF-3 and its derivatives are likely to diffuse poorly through some matrixes, including agar and paper.

### 3.3. MIC Values of DIF Derivatives in S. aureus, E. faecalis, and E. faecium

We then further assessed the potential antibacterial activities of representative DIF derivatives (Figure 1) on the growth of MSSA (strain ATCC29213), MRSA (ATCC43300), VSE (ATCC29212), and VREs (ATCC51299 and ATCC700221); the latter two strains carry the vancomycin-resistant genes *vanB* and *vanA*, respectively (Table 2). Again, several DIF derivatives showed strong antibacterial activity against MSSA and MRSA (*S. aureus* strains different from those in Table 1), with MIC values of less than 2 µM (Table 2). In addition, various DIF derivatives demonstrated strong antibacterial effects against VSE and two VRE strains, with associated MIC values of less than 2 µM (Table 2); note that the MRSA strain was highly resistant to cefoxitin and oxacillin, and the two VREs were highly or intermediately resistant to vancomycin and ampicillin (Table 2).

These results indicate that DIF derivatives may possess strong antibacterial activities against a broad range of Gram-positive bacteria, including known drug-resistant bacteria such as MRSAs and VREs.

### 3.4. Effects of DIF Derivatives on the Ultracellular Structure of S. aureus

**Figure 4:**
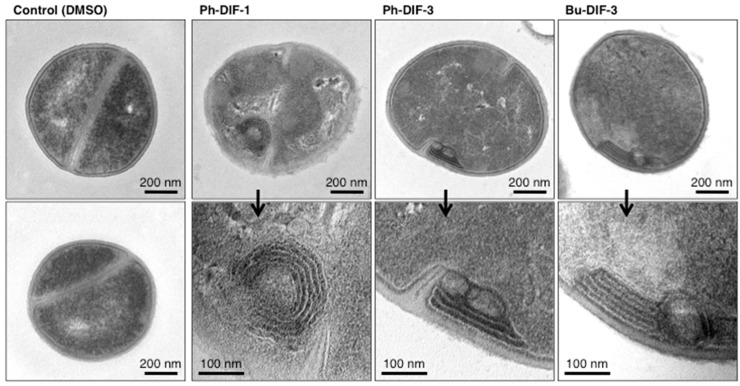
Effects of DIF derivatives on the ultracellular structure of *S. aureus* (MSSA). MSSA (ATCC29213) was incubated for 1.5 h at 37 °C in MHB in the presence of 0.2% DMSO (vehicle) or 4 µM of Ph-DIF-1, Ph-DIF-3, or Bu-DIF-3. The cells were collected, treated as described in the Materials and Methods section, and observed under a transmission electron microscope. Two representative photos of the DMSO-treated control MSSA and two representative photos of each DIF-treated MSSA are shown. The DIF-treated MSSA samples each show distinct multilayered structures composed of cell wall and/or plasma membrane.

To help elucidate the mechanism underlying the antibacterial activity of DIF derivatives, we examined the effects of Ph-DIF-1, Ph-DIF-3, and Bu-DIF-3 on the ultracellular structure of MSSA (strain ATCC29213). Transmission electron microscopy revealed distinct multilayered structures consisting of cell wall and/or plasma membrane in the DIF-treated MSSA cells (Figure 4). This finding suggests that the tested DIF derivatives suppress the growth of *S. aureus* by hindering cell wall formation.

### 3.5. Comparison of the Antibacterial Effects of Chlorinated Dibenzofurans and DIFs on S. aureus

**Figure 5:**
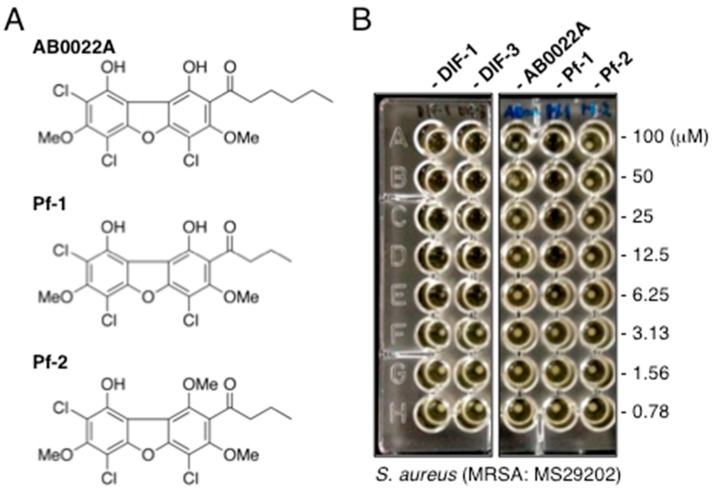
(**A**) Chemical structures of the chlorinated dibenzofurans AB0022A, Pf-1, and Pf-2. (**B**) Effects of chlorinated dibenzofurans and DIFs on the growth of *S. aureus* (MRSA). MRSA (MS29202) was incubated for 20 h at 37 °C in the presence of the indicated concentrations of DIF-1, DIF-3, AB0022A, Pf-1, or Pf-2, after which colony formation was noted for determining the MICs of the compounds.

The antibacterial substance AB0022A, a chlorinated dibenzofuran (Figure 5A), was isolated from *D. purpureum* [30] and is similar in structure to DIF-1. Like DIF-1 and several of its derivatives, AB0022A inhibits the growth of Gram-positive bacteria; its MIC values against *S. aureus* (strain IID671), MRSA (IID1677), *E. faecalis* (ATCC29212), and *B. subtilis* (ATCC6633) are 1.56 µg/mL (3.4 µM), 3.13 µg/mL (6.8 µM), 50 µg/mL (109 µM), and 0.78 µg/mL (1.7 µM), respectively [30], and thus are at least somewhat comparable to those of DIF derivatives (Table 1 and Table 2). In addition, two other chlorinated dibenzofurans, Pf-1 and Pf-2 (Figure 5A), have been isolated from the cellular slime mold *Polysphondylium filamentosum* [31], but their antibacterial activities have not previously been examined.

When we assessed the MIC values of these dichlorinated dibenzofurans in MRSA (strain MS29202), neither AB0022A nor Pf-2 had any noteworthy inhibitory effect (MIC, >100 µM) on the growth of this strain (Figure 5B). In contrast, Pf-1 suppressed the growth of this MRSA at a MIC value of 12.5 µM, which was comparable to those of DIF-1 and DIF-3 (25 and 12.5 µM, respectively). The results suggest that DIF derivatives such as CP-DIF-3 and Ph-DIF-3 (Table 1 and Table 2) are likely better lead antibacterial drugs than the chlorinated dibenzofurans we evaluated.

## 4. Discussion

Most of the many antibiotics (antimicrobials) identified since the discovery of penicillin in the fungus *Penicillium notatum* have been isolated from fungi and Actinobacteria [33,34,35,36]. However, due to intensive use, overuse, or the use of subtherapeutic concentrations of antibiotics, the number of drug-resistant bacteria, such as MRSAs and VREs, is growing. A search for new antibiotic molecules and bio-resources that produce novel antimicrobials is required [9,35,36,37].

The cellular slime mold *D. discoideum* has long been used as a model organism in the study of cell and developmental biology. However, slime molds have recently been identified as excellent sources of potential lead compounds for drug discovery and the development of novel medicines [9,38]. In this regard, we have focused on elucidating the biologic and pharmacologic activities (such as antitumor and antiparasitic effects) of the *D. discoideum*-derived DIFs and their derivative compounds in various types of eukaryotic cells [9,16,29].

In the present study, we showed that several *D. discoideum* DIF derivatives possess antibacterial activity against Gram-positive bacteria, including *S. aureus*, *E. faecalis*, *E. faecium*, and *B. subtilis*. In contrast, these DIF derivatives did not inhibit the growth of *Mycoplasma* or the Gram-negative bacterium *E. coli* (Table 1 and Table 2). Most importantly, several of the DIF derivatives we evaluated strongly suppressed the growth of MRSAs and VREs (Table 1 and Table 2). Note that the MRSA strain MS29202 was resistant not only to penicillin and streptomycin (Figure 2B) but also to clindamycin, levofloxacin, and imipenem (Figure 2C), whereas the MRSA strain (ATCC43300) was resistant to both cefoxitin and oxacillin (Table 2). In addition, the VRE isolates *E. faecalis* (which harbors the vancomycin-resistance gene *vanB*) and *E. faecium* (containing *vanA*) were resistant to both vancomycin and ampicillin (Table 2). Although they are primarily opportunistic pathogens, *E. faecalis* and *E. faecium* cause the great majority of enterococcal infections, and isolates that carry drug-resistance genes such as *vanA* or *vanB* can cause serious infections [39,40]. Overall, our results suggest that the mechanism underlying the antibacterial actions of DIF derivatives likely differ (at least in part) from those of the known antibiotics we assessed. Consequently, DIF derivatives are promising lead compounds for novel antimicrobials against a broad range of Gram-positive bacteria, including known drug-resistant strains.

Although the antibacterial activities of the DIF derivatives varied among the Gram-positive strains of bacteria examined in this study (Table 1 and Table 2), the structure–activity relationship analysis revealed that compounds with longer alkyl chains at the acyl group (e.g., DIF-3(+2) and DIF-3(+3)) and compounds with larger alkyl groups in place of the methyl group (e.g., Bu-DIF-3, Hex-DIF-3, and Ph-DIF-3) had greater antibacterial activities against Gram-positive strains than did those with shorter alkyl chains or smaller alkyl groups, and that compounds with one chlorine substituted in the benzene ring had greater antibacterial activities against Gram-positive strains than did those with two chlorines substituted in the benzene ring. In contrast, there was no clear overall relationship between hydrophobicity (assessed as ClogP value) and antibacterial activity (Table 1).

Regarding the mechanism through which DIF derivatives exert their antibacterial effects, DIF derivatives have mitochondrial uncoupling activity in mammalian cells [17,18]. Given that mitochondria and bacteria are similar in many aspects, these previous findings suggest that DIF derivatives may affect bacterial proton transport (or intracellular pH) and thereby suppress bacterial growth. However, the strength of the mitochondrial uncoupling properties of DIF derivatives [17,18] does not necessarily correlate with the strength of their antibacterial activities (Table 1 and Table 2). Furthermore, in the present study, we showed that in *S. aureus*, Ph-DIF-1, Ph-DIF-3, and Bu-DIF-3 induce the formation of distinct multilayered structures composed of cell wall or membrane (Figure 4). Although the relationship between the antibacterial effects of DIFs and the DIF-induced formation of these structures is unclear currently, this observation may be a clue for elucidating the mechanism underlying the antibacterial activity of DIF derivatives.

## 5. Conclusions

In this study, we showed that several derivatives of DIF-1 and DIF-3, chlorinated polyketides found in *D. discoideum* possess strong antimicrobial activities against Gram-positive bacteria including MRSAs and VREs. Our results suggest that the DIF derivatives are good lead compounds for developing novel antimicrobials.

## 6. Patents

The following authors hold a patent related to this article:

Kubohara, Y.; Kikuchi, H.; Oshima, Y. Antibacterial drugs. Japanese Patent No. 6478378, 15 February 2019.

## Figures and Tables

**Table 1:** ClogP and minimum inhibitory concentration (MIC) (µM) values of DIFs and DIF derivatives against MSSA, MRSA, *Bacillus subtilis*, *Escherichia coli,* and *Mycobacterium bovis*.

Compound	ClogP	*S. aureus*		*B. subtilis*	*E. coli*	*M. bovis*
		MSSA: 209P	MRSA: MS29202	ATCC6633	(NIHJ)	ATCC25523
DIF-1	3.46	12.5	25	12.5	>100	>100
DIF-3	2.90	25	12.5	12.5	>100	>100
Br-DIF-1	4.00	25	50	25	>100	>100
Br-DIF-3	3.18	25	50	25	>100	ND
I-DIF-1	5.06	ND	12.5	ND	ND	ND
I-DIF-3	3.70	50	100	100	>100	>100
TM-DIF-1	3.99	>100	>100	>100	>100	ND
TH-DIF-1	3.20	>100	>100	>100	>100	ND
TH-DIF-3	2.64	>100	>100	50	>100	ND
DIF-1(−2)	2.63	100	100	50	>100	ND
DIF-3(−2)	2.07	>100	>100	50	>100	ND
DIF-1(−1)	3.05	25	50	25	>100	ND
DIF-3(−1)	2.49	50	50	25	>100	ND
DIF-1(+1)	3.88	12.5	25	3.13 *	>100	ND
DIF-3(+1)	3.32	3.13 *	1.56 *	3.13 *	>100	ND
DIF-1(+2)	4.30	6.25	12.5	0.78 *	>100	>100
DIF-3(+2)	3.74	1.56 *	3.13 *	3.13 *	>100	ND
DIF-3(+3)	4.16	0.78 *	0.78*	3.13 *	>100	>100
DIF-1(3M)	3.43	12.5	12.5	6.25	>100	ND
DIF-3(3M)	2.87	12.5	25	12.5	>100	ND
DIF-1(CP)	3.11	12.5	12.5	12.5	>100	ND
DIF-3(CP)	2.74	100	100	25	>100	ND
Et-DIF-1	3.80	6.25	12.5	1.56 *	>100	ND
Et-DIF-3	3.24	3.13 *	3.13 *	3.13 *	>100	ND
Bu-DIF-1	4.70	>100	>100	0.39 *	>100	>100
Bu-DIF-3	4.15	0.39 *	0.78 *	1.56 *	>100	>100
Hex-DIF-3	4.98	1.56 *	0.78 *	3.13 *	>100	>100
CP-DIF-1	4.59	>100	>100	>100	>100	>100
CP-DIF-3	4.03	0.78 *	0.78 *	0.78 *	>100	>100
Ph-DIF-1	5.13	12.5	25	0.78 *	>100	>100
Ph-DIF-3	4.57	0.78 *	0.39 *	0.78 *	>100	>100

ClogP (hydrophobic index) and MIC values of the indicated DIFs and DIF derivatives were assessed as described in the Materials and Methods section. ND, not determined. * MIC values less than 5 µM.

**Table 2:** MIC (µM) values of DIFs and derivatives against MSSA, MRSA, VSE, and VRE.

Compound	*S. aureus*		*E. faecalis*		*E. faecium*
	MSSA	MRSA	VSE	VRE (VanB)	VRE (VanA)
	ATCC29213	ATCC43300	ATCC29212	ATCC51299	ATCC700221
DIF-1	6.25 ± 0	8.33 ± 3.6	41.7 ± 14.4	41.7 ± 14.4	41.7 ± 14.4
DIF-3	33.3 ± 14.4	25 ± 0	100 ± 0	>100	>100
DIF-1(+1)	16.7 ± 7.2	12.5 ± 10.8	25 ± 21.7	25 ± 21.7	25 ± 21.7
DIF-3(+1)	10.4 ± 3.6	6.25 ± 0	10.4 ± 3.6	37.5 ± 21.7	10.4 ± 3.6
DIF-1(+2)	6.25 ± 0	1.56 ± 0 *	3.13 ± 0 *	6.25 ± 0	3.13 ± 0 *
DIF-3(+2)	5.21 ± 1.8	4.17 ± 1.8 *	4.17 ± 1.8 *	54.2 ± 43.9	4.69 ± 2.7 *
DIF-3(+3)	2.61 ± 0.91 *	2.35 ± 1.36 *	1.56 ± 0 *	3.65 ± 2.39 *	1.56 ± 0 *
Et-DIF-1	25 ± 0	12.5 ± 0	33.3 ± 14.4	50 ± 0	33.3 ± 14.4
Et-DIF-3	5.21 ± 1.8	5.21 ± 1.8	18.8 ± 10.8	100 ± 0	>100
Bu-DIF-1	>100	1.56 ± 0 *	2.61 ± 0.91 *	14.6 ± 9.5	3.13 ± 0 *
Bu-DIF-3	2.35 ± 1.36 *	1.3 ± 0.45 *	1.56 ± 0 *	3.65 ± 2.39 *	1.56 ± 0 *
Hex-DIF-3	7.29 ± 4.77	1.3 ± 0.45 *	0.78 ± 0*	1.56 ± 0 *	1.17 ± 0.55 *
CP-DIF-1	ND	ND	100 ± 0	>100	>100
CP-DIF-3	1.3 ± 0.45 *	1.04 ± 0.45 *	1.56 ± 0 *	2.61 ± 0.91 *	1.56 ± 0 *
Ph-DIF-1	6.25 ± 0	1.3 ± 0.45 *	2.61 ± 0.91 *	2.61 ± 0.91 *	1.56 ± 0 *
Ph-DIF-3	3.65 ± 2.39 *	0.78 ± 0 *	1.56 ± 0 *	2.61 ± 0.91 *	1.56 ± 0 *
Cefoxitin	6.25	>100	ND	ND	ND
Oxacillin	0.2 *	>100	ND	ND	ND
Vancomycin	0.39 *	0.39 *	0.39 *	25	>100
Ampicillin	ND	ND	0.2 *	12.5	>100

MIC values of the indicated DIF derivatives and the indicated antibiotics were assessed as described in the Materials and Methods section, and mean values ± 1 SD were determined from three independent experiments. ND, not determined. * MIC values less than 5 µM.
